# How to increase our belief in discovered statistical interactions via large-scale association studies?

**DOI:** 10.1007/s00439-019-01987-w

**Published:** 2019-03-06

**Authors:** K. Van Steen, J. H. Moore

**Affiliations:** 1 WELBIO, GIGA-R Medical Genomics–BIO3, University of Liège, Liege, Belgium; 2 Department of Human Genetics, University of Leuven, Leuven, Belgium; 3 Institute for Biomedical Informatics, University of Pennsylvania, Philadelphia, USA

## Abstract

The understanding that differences in biological epistasis may impact disease risk, diagnosis, or disease management stands in wide contrast to the unavailability of widely accepted large-scale epistasis analysis protocols. Several choices in the analysis workflow will impact false-positive and false-negative rates. One of these choices relates to the exploitation of particular modelling or testing strategies. The strengths and limitations of these need to be well understood, as well as the contexts in which these hold. This will contribute to determining the potentially complementary value of epistasis detection workflows and is expected to increase replication success with biological relevance. In this contribution, we take a recently introduced regression-based epistasis detection tool as a leading example to review the key elements that need to be considered to fully appreciate the value of analytical epistasis detection performance assessments. We point out unresolved hurdles and give our perspectives towards overcoming these.

## Introduction

Genome-wide association studies (GWAS) have allowed for greater understanding of the genetic contributions to complex traits and the mapping of disease susceptibility loci. However, the initial paradigm of focusing a study on one SNP at a time or on a selection of candidate genes has limited our ability to identify novel genes underlying susceptibility to human disease. Likewise, it has not been efficient in the detection of genetic factors that may be depending on interactions with the other genes (epistasis) or environmental stimuli. The term “epistasis” was first described by William Bateson (1907), referring to the suppression of gene expression at one locus by a gene at another locus. Fisher’s definition of epistasis (Fisher 1918) indicates deviations from additivity in the effect of alleles at different loci with respect to their contribution to a quantitative phenotype, which was shown not to be equivalent to Bateson’s definition (Norton and Pearson 1976). Nowadays, the term “epistasis” is broadly used to refer to interactions between genes in which the contribution of one gene to a phenotype depends on the genetic background of the organism under study. A further distinction is made between biological epistasis and statistical epistasis: Biological epistasis [or physiological epistasis (Sackton and Hartl 2016)] can be seen as a result of physical interactions among biomolecules within gene regulatory networks and biochemical pathways in an individual (Moore and Williams 2005), whereas statistical epistasis assumes deviations from multi-locus additive genetic effects in a mathematical model for a phenotype. For more details about epistasis, its meaning, related concepts, and general discussions about analytic issues, we refer to educational work published since 2002, including (Moore and Williams 2005; Cordell 2002, 2009; Phillips 2008; Van Steen 2012; Wei et al. 2014; Ritchie and Van Steen 2018).

Even though the proportion of heritability that is due to epistasis for complex traits in humans remains hard to estimate to date [see for a discussion in (Sackton and Hartl 2016)], there is no doubt that epistasis plays a role in human complex genetics. One role is in personalized medicine where differences in biological epistasis may impact disease risk, diagnosis, or disease management [see, for instance, (Moore and Williams 2009), epistasis in drug resistance (Duraisingh and Refour 2005; Kim et al. 2011; Wilson et al. 2016), and epistasis in Alzheimer’s disease (Gusareva et al. 2014) with a discussion about biological translation (Ebbert et al. 2015)]. This understanding stands in wide contrast to the unavailability of a widely accepted protocol to perform a Genome-Wide Association Interaction Study (GWAIS). The lack of such a standardized detailed workflow can be explained by the many difficulties involved in performing large-scale epistasis screening and in inferring biological evidence from statistical findings (Gusareva and Van Steen 2014; Bessonov et al. 2015). One of these involves the selection of the best analytic model to maximize the detection of multiple epistasis signals with biological relevance. Notably, modelling or statistical testing only involves one component of an entire GWAI analysis protocol (Gusareva and Van Steen 2014). Far too often, GWAI analysis procedures related to data cleansing/quality control (QC), missing observations, population structure control, effect encoding, and multiple testing are simply taken over from the GWAS scene. However, too little SNP variation will hamper statistical GWAI power, too high (distant) LD may induce redundant epistasis (Moore et al. 2006), additive encoding schemes will elevate type I error rates and augment the number of false-positive GWAI findings, and complex dependencies between interaction test statistics will affect the performance of multiple testing strategies typically employed in the context of GWA studies (Gusareva and Van Steen 2014). Whether or not a (newly) introduced GWAI analytic strategy can be promoted over another strategy in real-life data applications will largely depend on the degree to which all of the above, as a minimum, is taken into account in formal performance assessments on synthetic data.

In this contribution, we take a recently suggested novel epistasis detection tool (Frånberg et al. 2015), based on the most commonly used regression paradigm, as a leading example, and suggest different contexts to help interpret performance assessments of large-scale epistasis screening approaches. There was no particular reason to have selected the work of Frånberg et al. (2015); there are ample publications on regression-based epistasis methods which could have equally likely served our purpose [e.g., BOOST (Wan et al. 2010), EPIBLASTER (Kam-Thong et al. 2011), FRGEpistasis using functional regression (Zhang et al. 2014) and epistasis detection with penalized regression (Slim et al. 2018)].

## Multi-stage epistasis screening: an example

In their paper, Frånberg et al. (2015) display an interesting novel multi-stage epistasis screening approach based on likelihood ratio tests derived from regression models. The idea is to increase the power of a GWAI study by investigating two strategies that can be combined. The first allows for flexible incorporation of different trait scales. The second aims to reduce the multiple testing problem via a series of hypothesis testing that involves simpler models than a full interaction model with two SNPs, say SNP1 and SNP2. In particular, the full model relates to HA: $g\left({p}_{12}\right)= \alpha + {\beta }_{1}{\text{SNP}}_{1 }+ {\beta }_{2}{\text{SNP}}_{2 }+ {\beta }_{12}{\text{SNP}}_{1 }*{\text{SNP}}_{2},$ in which ${p}_{12}$ represents the mean response conditional on SNP1 and SNP2 that are encoded as 0, 1, 2, and in which $g(.)$ represents an appropriate link function. The simpler models are the intercept-only model (H1: $g\left({p}_{12}\right)= \alpha )$, the main-effect models for SNP1 (H2: $g\left({p}_{12}\right)= \alpha + {\beta }_{1}{\text{SNP}}_{1 })$ and for SNP2 (H3: $g\left({p}_{12}\right)= \alpha + {\beta }_{2}{\text{SNP}}_{2 })$ and the additive model including both SNP1 and SNP2 in the absence of an interaction term (H4: $g\left({p}_{12}\right)= \alpha + {\beta }_{1}{\text{SNP}}_{1 }+ {\beta }_{2}{\text{SNP}}_{2 }$). Rather than performing the so-called conditional model building that would imply starting with no SNPs in the model, then testing the addition of each SNP separately or jointly in the model, and finally testing for interaction between SNP1 and SNP2, Frånberg et al. consecutively test Hi (*i* = 1,2,3,4) vs HA, in this order. In other words, it is the full model that is constantly being tested for against a variety of “null” models. The multi-stage procedure obviously comes with a corrective method to maintain type I error rates. Two such corrective methods are proposed: a so-called static one, in which, at each stage, a pre-estimated number of tests is used in a Bonferroni type of family-wise error rate (FWER) control, and a so-called adaptive one, in which the aforementioned estimate is replaced by the actual number of tests performed at each stage. The motivation behind their strategy is that when an SNP pair’s relationship to a trait can adequately be described by a simple model, there is no need to make the model more complex. In this case, the SNP pair can be excluded from the epistasis screening panel, hereby relaxing the computational and multiple testing burden.

The aforementioned work involves one of the few synthetic data studies that considers the inclusion of a large variety of penetrance tables since the publication of Li and Reich in (2000). Two-locus models for biallelic SNPs are typically represented by three-by-three penetrance tables. Each entry (“penetrance”) refers to the probability of being affected with disease, for a specific (1 out of 9) 2-locus genotype combination. The assumption of full penetrance (i.e., all entries in the penetrance table are either 1 or 0) is relaxed via controlling the total heritability due to the two loci under investigation (Culverhouse et al. 2002). In addition, Frånberg et al. (2015) are among the first to propose and evaluate a test statistic that is invariant to a variety of scales relating genotype to phenotype, showing the expected power advantages of their method over some mainstream analyses based on a regression framework. The latter is important as it is a known fact in epidemiology that interactions may appear or disappear depending on the measurement scale used to describe the outcome of interest (Greenland and Rothman 1998).

## How to evaluate a novel epistasis screening tool?

**Fig. 1:**
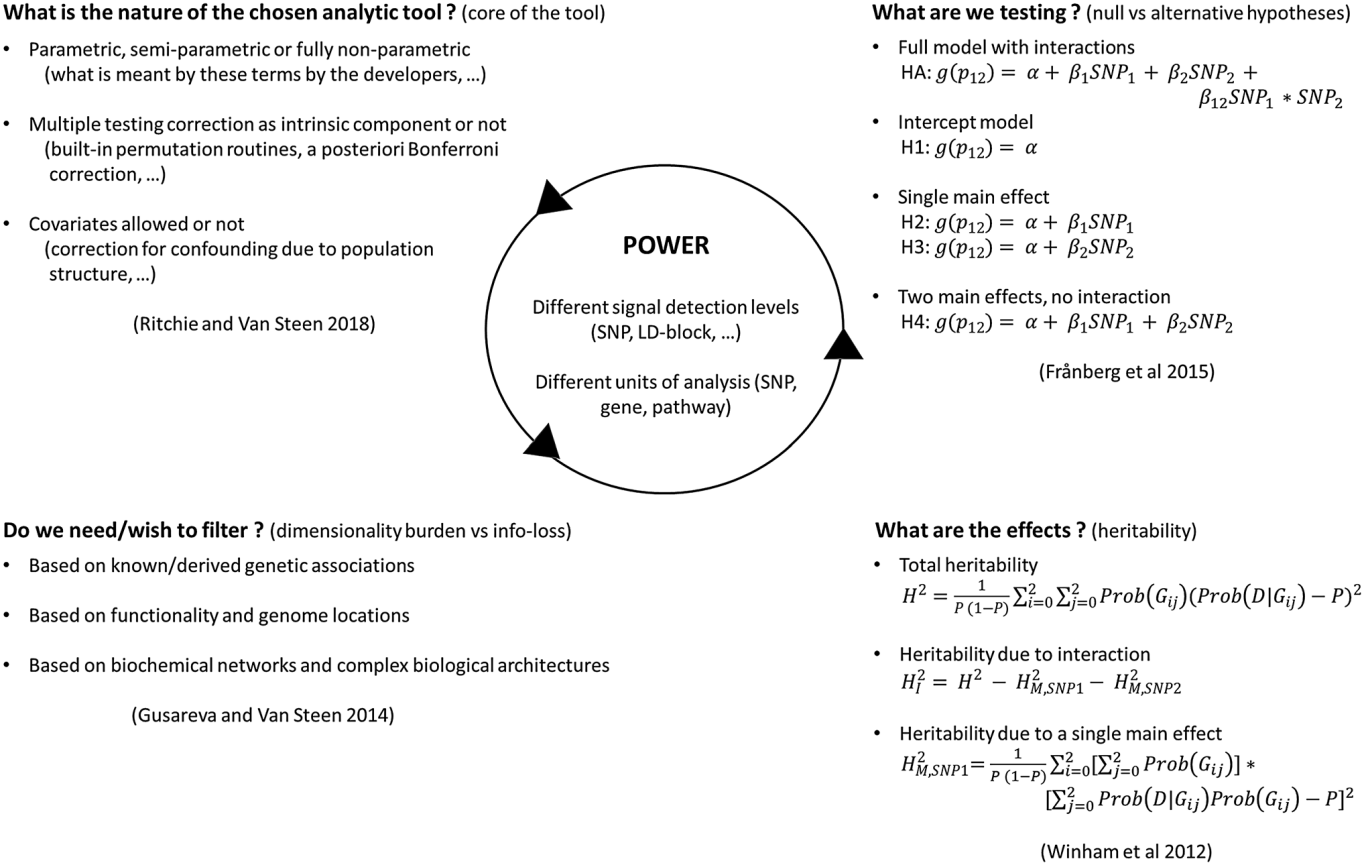
Minimal number of attention points to consider when evaluating a novel analytic tool on real-life or synthetic data. All of these affect power, which can be defined at different levels and may involve interpreting analysis results derived from SNPs, genes, or pathways as units of epistasis analysis

Some attention points are worth mentioning when evaluating a novel epistasis detection method (see Fig. 1). These relate to setting up a comprehensive simulation study as well as interpreting its results and assessing the impact of the method for the community.

### Heritability and frequency spectrum of genetic variants

In Frånberg et al.(2015), for a pre-specified heritability, non-zero penetrances were set to the same value, and power results were summarized over all the considered models, without distinguishing between purely epistatic models or epistasis models with main effects. In fact, Frånberg et al. use total heritability ${H}^{2}$ as a summary measure of all the genetic effects in a two-locus model, with ${H}^{2}$ defined as follows: ${H}^{2}=\frac{1}{P (1-P)}\sum _{i=0}^{2}\sum _{j=0}^{2}\text{Prob}\left({G}_{ij}\right){(\text{Prob}\left(D|{G}_{ij}\right)-P)}^{2}$ and $\text{Prob}\left({G}_{ij}\right)$ the frequency of the 2-locus genotype combination SNP1 = *i* and SNP2 = *j* (*i,j* = 0,1,2), and $P$ and $\text{Prob}\left(D|{G}_{ij}\right)$ denoting the disease prevalence and penetrance of the disease, respectively. However, quantifying the strength of simulated marginal and interaction effects in terms of heritability gives an added value as it contributes to a better assessment of the relative importance of main and interaction effects in the data. For instance, following (Winham et al. 2012), the heritability ${H}_{I}^{2}$due to the interaction effect of SNP1 and SNP2 can be defined as the portion of ${H}^{2}$ that cannot be attributed to the marginal effects of SNP1 and SNP2: ${H}_{I}^{2}= {H}_{}^{2}- {H}_{M, \text{SNP}1}^{2}- {H}_{M, \text{SNP}2}^{2}$ in which the heritability ${H}_{M, \text{SNP}1}^{2}$due to SNP1 only can be defined as ${H}_{M, \text{SNP}1}^{2}=\frac{1}{P (1-P)}\sum _{i=0}^{2}[\sum _{j=0}^{2}\text{Prob}\left({G}_{ij}\right)\left]\right[\sum _{j=0}^{2}{Prob\left(D|{G}_{ij}\right)\text{Prob}\left({G}_{ij}\right)-P]}^{2}$, and the heritability ${H}_{M, \text{SNP}2 }^{2}$due to SNP2 alone can be defined similar to ${H}_{M, \text{SNP}1}^{2}$. These definitions imply that the actual heritability due to the interaction may be much smaller than the lowest total heritability of 0.005 that Frånberg et al. considered for their synthetic data generation. This probably explains the large cases-plus-controls sample sizes of 4000, 6000, and 8000 included in their study to achieve appreciable power, with any of the considered analytic epistasis detection strategies. Notably, realistic scenarios can be formulated directly at the trait level (i.e., in terms of expected trait values) or at the variance scale (i.e., in terms of variance decomposition). The two formulations are equivalent, yet there is a tendency for the trait-level formulation (penetrance tables and risk of affection) to be omnipresent in case–control studies and the variance decomposition formulation in quantitative trait settings. The latter formulation explicitly allows one to control the proportion of epistatic and environmental variance to total genetic variance (Mahachie John et al. 2011a).

### Variable selection and filtering

Key to the outcome of any GWAI screening is the a priori variable selection step. For instance, Frånberg and colleagues (Frånberg et al. 2015) assume three such variable selection schemes on their real-life data application using the adaptive version of their multi-stage approach: no selection, selection based on main effects evidence from GWAS, selection based on significant interactions via omics experiments. Care has to be taken when considering main effects resulting from classic GWA studies into the multi-stage strategy. These main effects are the result of H2/H3 vs H1 testing, which is different from H2/H3 testing vs HA in (Frånberg et al. 2015). A plethora of variable selection approaches exist, not to be confused with variable extraction approaches (Van Steen 2012). Whereas the last typically involves a change in the representation of the original variables via aggregation or projection steps (such as principal components), the first simply involves selecting a subset of the original variables for subsequent analyses. Machine learners further make a distinction between those variable selection methods that (a) choose relevant subsets independent of the subsequent analysis method (aka filter methods), (b) choose subsets of variables as part of the learning procedure (aka embedded methods), or (c) perform variable selection according to their usefulness to a given predictor (aka wrapper methods). Examples of, respectively, filter, embedded, and wrapper methods include (a) significant main effects or nearest-neighbor-based filtering (Gola and König 2016), (b) regression with regularization (Park and Hastie 2008), or (c) stepwise regression (Guyon and Elisseeff 2003). More specifically, in the context of large-scale epistasis screening, reducing the computational complexity by filtering the genetic variants to a smaller subset with prior biological knowledge or by pre-processing the data using any statistical or computational method to identify those variants with the highest likelihood of being associated with the endpoint can be advantageous (Sun et al. 2014). One of the more promising approaches is based on the Relief family of machine-learning algorithms (Moore 2015) that are able to weight variants involved in non-additive interactions without performing a combinatorial search. The Spatially Uniform ReliefF (SURF) method was designed specifically for modelling epistasis (Greene et al. 2009a) and has been used in conjunction with approaches such as Multifactor Dimensionality Reduction (MDR) (Collins et al. 2013). However, there is also a price to pay when adopting filtering algorithms: they may eliminate true causal variants from the search pool. For additional examples of filtering approaches in the context of epistasis, and a discussion, we refer to (Van Steen 2012).

Often observed, as was also the case for the real-life data application in (Frånberg et al. 2015), little overlap exists in significant epistasis findings derived from different a priori variable selection procedures. Though, regardless of whether a priori knowledge is used or not as part of the GWAI protocol, the actual underlying biology of the problem never changes. Hence, how to interpret these differences? Which selection procedure best fits the underlying biological truth? Bessonov et al. (2015) made an attempt to answer such questions. Using prior biological information seemed to give more overlap in statistical epistasis findings between the GWAI protocols applied to real-life data compared to exhaustive GWAI protocols, suggesting that, if the prior information is reliable and there truly is epistasis in line with this prior knowledge, the alternative hypothesis space is narrowed down and genuine epistasis effects are more likely to be highlighted by different analysis strategies or GWAI protocols. Also in (Bessonov et al. 2015) (see Fig. 2 therein), no overlap was found between exhaustive and non-exhaustive screening protocols, suggesting that a large number of epistasis findings may get lost in the multiple testing correction, the latter being a correction with no biological connotation. In turn, when looking at multiple testing strategies that potentially generate additional significant results when more SNPs are added (such as permutation-based MaxT as part of MB-MDR (Van Lishout et al. 2015), in contrast to Bonferroni correction in combination with the BOOST regression models (Wan et al. 2010), it seems that some significant epistasis findings via exhaustive screening protocols should be genuine, but may never be identified via restrictive searches in the way that these are currently being performed (Bessonov et al. 2015).

### Intrinsic properties of the chosen modelling paradigm

Frånberg’s choice to build a screening strategy on the regression paradigm is understandable because of its ease of implementation and wide-spread use. However, regression modelling in GWAIS comes at a cost. Often, there is no direct correspondence between the interaction effects in regression models and underlying penetrance tables representing epistasis effects (North et al. 2005). Relevant regression parameter estimates and derived interaction tests are typically not robust to model misspecification and high-dimensional confounding (Vansteelandt et al. 2008, 2012). It can give rise to inflated false-positive rates and reduced power, caused by the presence of sparse data and multiple testing issues, even in small simulated data sets only including 10 SNPs (Vermeulen et al. 2007). Furthermore, interaction terms in regression modelling can interchangeably be interpreted as effect modifier terms, whereas “interactions” and “effect modification” can be shown to be conceptually different (VanderWeele 2009; Knol and VanderWeele 2012). Some of the aforementioned problems are exacerbated when high-order interactions (order > 2) are the target.

To deal with some of the aforementioned issues related to the classic regression framework, and aiming to further improve the power of gene-interaction studies with SNPs, Hastie and Park (2008) proposed a penalized regression strategy: a variation on logistic regression with a size constraint on the *L*_2_-norm of the regression coefficients (excluding the intercept). Quadratic regularization overcomes collinearity among the SNPs, and thus, high-order interactions are easily accommodated. In their approach, each SNP level is coded by a dummy variable (binary encoding) with direct interpretation. Variables are selected using a forward stepwise procedure based on a cost-complexity statistic. Interaction terms may enter the model in the presence of only one constituent variable [i.e., asymmetric hierarchy (Friedman 1991)]. To avoid large main effect coefficients to be broken into sums of small interaction components, particularly for large values of the quadratic penalty parameter, the authors suggest to use a forward stage-wise variable selection procedure, only penalizing the coefficients for the newly selected variable. Although the penalized logistic regression approach was shown to outperform non-regression-based methods such as MDR (Ritchie et al. 2001), especially in the presence of multiple sets of significant SNPs, the strategy was never widely used by the epistasis community, in contrast to MDR. One explanation is the focus of the epistasis community on pairwise SNP interactions (mainly a pragmatic decision due to limited numbers of samples), for which the benefits of penalized logistic regression over classic logistic regression may be less pronounced. Another explanation is the extra-computational costs involved in tuning the penalization parameter, especially when based on cross-validation procedures and considering genome-wide screening contexts. There is some noteworthy similarity between the penalized logistic regression approach of Hastie and Park (2008) and the strategy proposed by Frånberg et al. (2015): both involve a component of testing for parsimony, where an interaction term is accepted in the model if one or both components are already in the model and if it provides a better fit to the data. As Hastie and Park state, the implemented penalized regression framework can be further relaxed to include interaction terms, although this was never implemented nor formally evaluated. Such an asymmetric hierarchy would be in contrast to the hierarchical model building in (Frånberg et al. 2015).

### Power estimation

Achieving consistent power over a variety of epistasis models is important as different SNP pairs may have different underlying genetic epistasis architectures, all of which we would like to identify. Power exceedance plots can be used to visualize the power performance of a method. In such plots, the *x*-axis refers to thresholds for power to detect epistasis and the *y*-axis refers to probabilities of the method’s power exceeding a threshold. The power exceedance plots depicted in (Frånberg et al. 2015) seem to indicate that, with consecutive testing of H1 vs HA, H2 vs HA, H3 vs HA and H4 vs HA, and adopting scale-invariant test statistics, consistent power advantages can be obtained. Which of the multi-stage approach or the scale-invariant test statistic is most responsible for explaining these advantages remains unclear. Exceedence plots are not widely used in epistasis contexts. A possible reason is the computational burden involved in creating them. The latter might also explain why a less computational expensive multi-stage approach than the advocated adaptive approach was considered for these plots in (Frånberg et al. 2015). On a side note, the proposed scale-invariant statistic by Frånberg et al. (2015) is based on the intersection–union principle which computes the final *p* value of H4 vs HA testing as the maximum of *p* values over different scales. It remains debatable which outcome transformations to consider and/or whether it is reasonable to allow that a single transformation, not leading to a rejection of H4, removes the SNP pair as pointer towards possible biological epistasis. Several scale transformations for binary outcomes can be considered, such as the entire Guerrero and Johnson (1982) family of power transformations, which can be seen as an extension to dichotomous outcomes of the Box–Cox power transformation family for continuous outcomes (Box and Cox 1964). Alternative to deciding upon which transformations to consider in the aforementioned scale-invariant stage-wise approach, it may be worth considering direct testing for removable SNP × SNP interactions, where a removable interaction is one that can be eliminated from the regression model by appropriate Guerrero and Johnson transformation of the outcome (Satagopan and Elston 2013). Only those pairs that do not withstand the first stage test for removable interaction are eligible for the second-stage interaction testing. How to optimally balance between type I and type II errors in this context warrants further investigation.

The power simulation settings considered in (Frånberg et al. 2015) not only assume fixed (static) stage weight corrections but also do not involve the generation of null SNPs. Actually generating null SNPs with variable minor allele frequencies (MAFs) would distinguish between power and “specific power”. Specific power would refer to the probability to identify the actual causal SNP pairs and no other pairs (Cattaert et al. 2011), whereas power refers to the probability to identify the actual causal SNP pairs, possibly supplemented with false-positive interaction pairs. To minimize costs related to experimental validation studies, it is essential to have clear views about the false positives generated by a GWAI strategy, apart from being able to identify the true causal SNP pair(s).

When evaluating the performance of epistasis tools, it is always good to look at the range of MAFs considered in the simulation studies. For instance, was it fixed at 0.03 (Frånberg et al. 2015) or were MAFs allowed to vary within [0.01–0.50]? Were causal SNP pairs assumed to have equal or different MAFs? Whereas, in classic GWA contexts, the minimum MAF is typically as low as 0.01, in GWAIS, it is often bounded below by MAF = 0.05 (Gusareva and Van Steen 2014). The latter is to avoid that there are too high numbers of multi-locus genotype combinations with no or very limited number of observations, jeopardizing large-sample statistics properties. Notably, Frånberg et al. aim to achieve the same by considering SNP combinations for which the “product of MAFs” is less than 0.04 in their real-life data application; a rather unconventional threshold. Under the assumption of locus independence, this threshold gives rise to a minimum probability of 0.0016 to observe an individual with two copies of the minor allele at two loci. Only a handful of studies explore the performance of tools for low-frequency variants (i.e. 0.01 $\le$ MAF  $\le \hspace{0.17em}$0.05) or rare variants. For low-frequency variants, for instance, the W test particularly performs well (in terms of power, non-specific) under scenarios of weak or moderate (i.e., 0.2 < LD *r*^2^$\le$ 0.8) and high LD (i.e., LD *r*^2^ > 0.8) (Wang et al. 2016). However, these authors did not reveal data on specific power, nor about the percentage of replicates in which the causal SNP pair was top ranked. This is a pity, especially in view of the LD-dependent scenarios which they considered. For rare variants, an exploratory study suggested a dramatic increase in false-positive epistasis findings when adopting epistasis detection strategies based on MDR-inspired dimensionality reduction (Mahachie John et al. 2011b). Binning and aggregation within a genetic unit (usually a gene), as adopted in rare variant exome analyses, may offer a solution (Fouladi et al. 2015), though more work is needed to better deal with the often unacceptable type I errors observed in rare variant association testing (Dering et al. 2014). Similar concerns are likely to apply for GWAIS that involve uncommon variants. Most likely, integration over multiple omics resources are needed to obtain genetic unit aggregates while maintaining adequate type I error control and acceptable power in large-scale whole-genome epistasis studies (Van Steen and Malats 2015). Lessons learned from rare variant association testing include that the selection of omics features to include in the aggregates will be critical (Zhang 2015).

Prior to looking at power performances of a strategy, it is advisable to investigate the strategy’s ability to control type I error. The static stage-wise method of (Frånberg et al. 2015) appears to have a tendency of being over-conservative, compared to the other considered modelling approaches based on logistic regression or MB-MDR and including the adaptive stage-wise method of Frånberg and colleagues. Using Bradley’s liberal criterion of robustness (Bradley 1978) [which states that the empirical Type I error rate should be in the interval (0.025, 0.075) under the assumption that the true significance level is 0.05], the adaptive strategy is still overly conservative in the absence of any association, in the presence of a single main effect, and in the presence of two main associations with log or logit links. Interestingly, in the realistic scenarios with multiple main effects (10, 20, or 30 main effects) additively impacting disease risk on a logit scale, the adaptive stage-wise approach of (Frånberg et al. 2015) applied to 4000 cases and 4000 controls is overly liberal (empirical type I error > 0.13). Note that the adaptive strategy was shown to adequately control FWER under “asymptotic conditions”. In addition, restricting attention to SNPs with MAFs ≥ 0.2 may be too restrictive, as some epistasis strategies such as W test and MB-MDR perform well for MAFs as low as 0.05 and sometimes even as low as 0.01 for particular simulation scenarios (Wang et al. 2016). The question thus emerges whether the, sometimes, liberal behaviour of the adaptive approach may elevate false-positive epistasis findings in real-life data applications. Finally, in (Frånberg et al. 2015), the highest type I error rates for logistic regression-based and MB-MDR testing were observed for the simulation models that link disease probability (and even more severe, disease odds) directly to two main effects in an additive way. This is not surprising as both logistic regression testing and MB-MDR for case–control designs involve the inclusion of main associations for outcomes considered on a logit scale. In addition, for fixed main effects parameters in a linear predictor, the proportion of the outcome variance explained will be highly dependent on the outcome scale.

### Multiple testing corrections

Multiple testing corrections are essential to keep type I errors under control, yet may negatively impact the power of a GWAI study (Bessonov et al. 2015). Despite the assertions of the authors, the multiple testing burden employed in (Frånberg et al. 2015) can be considered to be more expensive than in the initial setting of exhaustively testing for interactions, as the number of H1 vs HA tests coincides with the number of SNP pairs to interrogate, and such testing only involves the first stage of the four-stage GWAI procedure. Testing H1 vs HA is more powerful than testing H4 vs HA, yet it may also falsely point towards the full interaction model. This may be the case in the presence of strong main effects, and thus, testing H1 vs HA should be interpreted merely as evidence towards a more complex model than the intercept-only model. Often, choices about multiple testing but also choices about variable selection schemes or interaction testing itself are driven by pragmatism due to the inadequacy of available IT infrastructure and, thus, the inability to implement too computational intensive strategies. This may lead to choosing suboptimal GWAI analysis protocols. Ongoing efforts to increase the computational efficiency of approaches should diminish making such choices in the future. For instance, some genome-wide testing approaches for epistasis are particularly suitable for implementation on graphics processing units (GPUs) as powerful parallel processing units (Greene et al. 2010; Gonzalez-Dominguez et al. 2015; Putz et al. 2013; Kam-Thong et al. 2012; Hu et al. 2010). Not all hardwares are equipped with the necessary GPUs though in which case cloud computing (Wang et al. 2011) may offer a way out. However, cloud computing infrastructure does not offer unlimited resources, and thus, in some cases, hardware-oriented solutions, such as those based on FPGA architecture, may be worthwhile to look into, when possible (Gundlach et al. 2016). Although this would definitely help to make permutation-based minP multiple testing strategies feasible for GWAIS, it does not resolve the need to develop statistically more refined multiple testing correction techniques, which better take into account complex dependencies between all the performed tests. Inspiration can be retrieved from GWAS and Linkage Analysis practices where a variety of multiple testing methods have been developed that can accommodate correlated hypothesis testing, e.g., using the concept of effective sample size (Nyholt 2004; Moskvina and Schmidt 2008), by exploiting haplotype block structure (Nicodemus et al. 2005), or via adaptive exploitation of the dependence structure among hypotheses (Sun and Cai 2009), among others. In either case, to be able to make a complete assessment of the practical utility of a new GWAI screening method, minimal knowledge about (estimated) computation times with the tool, in a variety of settings, is essential.

### Linkage disequilibrium

Linkage disequilibrium (LD) in ancestral and admixed populations are key parameters driving the inconsistent regression models (Martin et al. 2018). Although the considered synthetic data sample sizes in (Frånberg et al. 2015) are becoming increasingly available in real life, samples of such sizes most likely cannot be assumed to be homogeneous. In fact, large- or fine-scale population structure (for instance due to differences in genetic ancestry) may be present. This structure needs to be accounted for in the association analyses to avoid increased false positives. In addition, structural differences between populations may be highly complex and non-linear (Alanis-Lobato et al. 2015). Whereas a modest number of linear PCs (typically ≤ 10) are sufficient to handle confounding in GWAS due to shared genetic ancestry, non-linear corrections may be necessary in GWAIS [work in progress—(Abegaz et al. 2018)].

LD between markers may not only induce dependencies between epistasis test hypotheses, it may also give rise to multicollinearity in regression-based approaches. Such multicollinearity can be defined as the existence of high multiple correlation when one of the variables is regressed on the others (Belsley et al. 1980). Basically, the source of multicollinearity can be sample-based or structural (Dohoo et al. 1996). The last source refers to multicollinearity induced after data collection, for instance, by introducing power terms in regression models. This form of multicollinearity is easily tackled, by mean-centering variables before taking powers (Glantz and Slinker 1990). The first source refers to the collected variables themselves being correlated. These correlations can comprise biologically meaningful dependencies or they can be an artefact of the acquired sample. Although multicollinearity can be seen as a data problem rather than a statistical problem, it may complicate statistical analyses. Indeed, the inclusion of SNPs with non-negligible LD in regression models may give rise to harmful multicollinearity (e.g., incorrect regression parameter estimates with large standard errors, leading to deflated test statistics) (Van Steen and Molenberghs 2012). Thus, special attention is required when testing H4 vs HA. For this reason, developers of novel epistasis strategies often assume no LD between markers in their synthetic data analyses. In addition, Frånberg et al. assumed markers to be at least 1 Mbp away from each other in their real-life data analysis. The latter does not necessarily remove highly correlated SNPs in the sample. In general, more elaborate approaches exist to prune genetic marker panels based on LD. Often such pruning is performed via sliding windows, as implemented in the PLINK software (Purcell et al. 2007). Again, long-distance correlations may still exist and may cause redundant epistasis or deflated/inflated parameter variance estimates leading to erroneous model selection. Some epistasis detection tools explicitly use LD to enhance power, building upon the assertion that interaction between two loci can create different LD patterns in disease and control populations (Wang et al. 2010). Examples of such tools include the fast-epistasis module in PLINK 1.9 (Purcell et al. 2007), EPIBLASTER (Kam-Thong et al. 2011), iLOC (Piriyapongsa et al. 2012), and the adjusted Wu test as implemented in CASSI (Ueki and Cordell 2012). LD may also impact multiple testing procedures as it may induce complex dependencies between tested hypotheses. For instance, the popular Bonferroni control of FWER (${\alpha }_{\text{Bonf}}= \frac{\alpha }{m}$, with *m* the total number of hypothesis tests and $\alpha$ usually set to 0.05) can be quite conservative for large numbers of possibly dependent tests, at the cost of increasing the number of false negatives (Goeman and Solari 2014). Positive dependent tests, as defined by Benjamini and Yekutieli (2001), may be induced by LD between markers, and thus, FDR control may be more appropriate than FWER control. Unfortunately, FDR methods typically start to break down in large-scale GWAI settings with the complex LD dependencies between markers. Even in GWAS, the power over Bonferroni is minimal, especially when multiple signals are assumed to be present in the data and the aim is to identify them jointly (Van Steen et al. 2005). Computationally demanding permutation-based approaches that account for multiple testing, such as the Westfall and Young step-down maxT implemented in (Van Lishout et al. 2013), can guarantee the strong control of the FWER under the subset pivotality assumption and weak control otherwise (Westfall and Young 1993). Subset pivotality may be jeopardized by interdependencies between markers. Weak control, referring to control of the Type I error rate under the complete null hypothesis only (i.e. all nulls true), may not be satisfactory. Hence, it is no surprise that—to date—there is no consensus about the most advantageous LD pruning threshold, balancing between optimal power and adequate false-positive control (Gusareva and Van Steen 2014). As a direct consequence, more work is needed to adequately capture pairwise interactions within a gene, as also these type of interactions may be of biological or clinical importance (Jorgenson and Witte 2006; Lehner 2011).

### Epistasis versus two-locus effect

A central challenge of any statistical or computational modelling approach is determining whether a significant result is due to additive effects, non-additive effects, or both. This can sometimes be difficult to disentangle for methods such as machine learning. One approach is to use the entropy-based methods to measure how much information about the endpoint is due to the synergistic effects of the variants after subtracting the marginal effects (Moore and Hu 2015). Another approach is to use permutation testing to specifically test the linear null hypothesis of additive effects. Such an explicit test for epistasis can be realized by randomizing the genotypes for each variant independently, within cases and controls separately, such that the interactions are disentangled but the genotype frequencies and thus the marginal effects are preserved (88). This approach has been extended and applied to machine-learning methods such as random forests (Li et al. 2016).

## From replication and validation toward translation

All of the above may complicate replicating GWAI results, especially at the SNP level (Gusareva and Van Steen 2014). Epistasis models can be highly sensitive to changes in allele or genotype frequency across data sets derived from different populations or across data sets that differ due to sampling inconsistencies (Greene et al. 2009b). In fact, power to replicate a genetic association can drop from more than 0.80 to less than 0.20 over a very narrow range of allele frequency change. This can greatly complicate the reproducibility of epistasis results. One approach is to resample the subjects from the replication data set, such that the multi-locus genotype frequencies match those from the detection data set. Such resampling approaches can dramatically improve the power to replicate epistasis results (Piette and Moore 2017), but need to be explored in more detail. Changing pre-selection schemes of markers (e.g., using prior biological knowledge), LD pruning or multiple testing criteriums, apart from the actual test statistics, can further impact the GWAI analysis results (Bessonov et al. 2015).

How important replication may be as a gold standard, even when evidence of replication in independent cohorts can be established (Evans et al. 2011), it may not be clear whether it really concerns a true positive or merely a false-positive replication (Bessonov et al. 2015). False-positive replication of epistasis above and beyond main effects may be induced by adopting the GWAI analysis protocols that cannot well differentiate between strong main effects and joint two-locus effects. Careful reflection should be given to choosing between hypothesis-free or hypothesis-driven GWAI screening, especially when adequate IT infrastructures are available. Both choices may give rise to non-overlapping epistasis findings, whereas the underlying biological context obviously remains the same. In contrast, when markers are filtered using reliable prior biological knowledge (Ritchie 2011), hereby narrowing down the alternative hypothesis space of possible epistasis models, chances may improve to see increased overlap between various GWAI workflows (Bessonov et al. 2015). Furthermore, pilot research seems to indicate that the impact of inadequate correction for population structure, potentially non-linear in nature, may be much more devastating in terms of increased false positives

and type I errors for GWAIS as compared to GWAS (personal communications). This imposes extra-analytic challenges and inhibits reproducibility. Whether or not gene-based epistasis can facilitate the reproducible identification of epistasis findings needs to be shown: It is often forgotten that confounding due to shared genetic ancestry or population admixture can act at both the SNP level and aggregated gene level (de Leeuw et al. 2016).

The path from genome to clinical outcome is characterized by complex wiring and cascades of actions and interactions. The presence and relevance of a potential biological gene–gene interaction, identified via statistical SNP × SNP interaction pointers, can only be verified via the experimental follow-up. This may include transcriptome analysis to investigate the co-expression of genes in particular tissues, immunofluorescence and confocal microscopy to confirm the presence of gene pairs in specific human cells as well as their colocalization in common cellular compartments, immunoprecipitation analysis to confirm physical interaction between genes in real biological systems, protein docking, and molecular dynamics experiments to increase insights into the actual mechanisms of the physical gene interactions (Gusareva et al. 2018). Validation procedures may also rely in part on a systematic epistasis literature review and structured knowledge from databases that integrates data from a variety of experimental platforms: e.g., cytoscape giving the integrated models of biomolecular interaction networks (Shannon et al. 2003), ConsensusPathDB using an integrated collection of molecular interactions in humans and model organisms (Shannon et al. 2003), GeneMANIA allowing to assess functional gene similarity via genomics and proteomics (Montojo et al. 2014), BioGraph for unsupervised biomedical knowledge discovery via automated hypothesis generation (Liekens et al. 2011), and IMP considering functional contexts of gene–gene networks from multiple organisms (Wong et al. 2015), to name but a few. Alternatively, methods are developed that lead to the improved biological insights, as is expected by integrating omics data or by building statistical epistasis networks from GWAI results. In such networks, nodes represent genes and (weighted) edges represent (the strength of) statistical gene–gene interactions (Lareau and McKinney 2015). The approach ideally assumes SNP-to-gene conversions or having aggregated information about a gene (Fouladi et al. 2015). It is a promising approach as it allows the detection of higher order (> 2) interactions by closely investigating genetic attributes that cluster together in the network (Hu et al.2013b). At the same time, it enhances the interpretation of statistical GWAI findings via exploitation of advanced network visualization tools (Hu et al. 2013a). In addition, networks are particularly handy for data integration purposes (e.g., by overlaying a statistical epistasis network with gene expression or protein interaction networks), or to investigate influential distorting factors and conditions of network instability or directionality (e.g., microbiome perturbations to human genome and interactome and causal network methodology).

GWAI findings obtained via statistical thinking may be hard to replicate, interpreting them, and thus, linking them to biological relevant processes is at least as tough. Part of the problem is related to the fact that different meanings have been given to epistasis over time. Compositional epistasis is said to be present when the effect of a genetic factor at one locus is masked by a variant at another locus and, therefore, links to the traditional definition of epistasis (Phillips 1998). The definition of epistasis being used in modern systems biology coincides with an extended version of compositional epistasis (Phillips 2008). Unfortunately, compositional epistasis is not equivalent to the presence of an interaction in a statistical model, but formal testing frameworks exist to detect it (VanderWeele and Laird 2011). Another part of the problem relates to the underdevelopment of translational tools that can be applied to GWAI results. For example, in GWAS, it is common to assist the interpretation of findings using enrichment methods that focus on genes in pathways. For GWAIS, restricting attention to gene sets linked to SNPs that appear in a list of top (statistically significant) SNP × SNP interactions seems to be limiting, as it does not directly use the discovered pairwise epistasis signals and most often uses distance-mapping rather than functional mapping of SNPs to genes. However, turning to statistical epistasis networks and analyzing those using network theory have been shown to increase interpretability (see before). Alternatively, combined network analysis and gene-interaction enrichment strategies (Liu et al. 2012) may be adapted to facilitate GWAI interpretation. In general, effective protocols are needed to help elucidating the functional repercussions of epistasis (Gusareva et al. 2014; Ebbert et al. 2015).

## Closing remarks

Because of the complexity of the problem being tackled, different viewpoints and analytic GWAI approaches exist. There is a need to further invest in understanding which strategies are best able to highlight particular genetic epistasis architectures, so as to develop ensemble approaches with optimal performance in large-scale genome-wide epistasis screening studies. This process would be greatly accelerated by making reference synthetic data sets available to the scientific community; data sets that are rich enough to embrace different degrees of complexity observed in real-life data, such as pathway interactions, non-genetic interferences, (non-linear) associations, and fixed and random dependencies between samples. The generation of such simulated data, for which the ground truth is known, is central to the development of new statistical and computational methods for detecting and characterizing epistasis. Data sets of minimal complexity can be obtained using parametric statistical models with a defined interaction term of a certain effect size. They can also be obtained using penetrance functions that define the probability for disease for different multi-locus genotype combinations that minimize marginal effects. Several methods and software have been developed for this purpose including the Genetic Architecture Model Emulator for Testing and Evaluating Software (GAMETES) that is also able to simulate genetic heterogeneity (Urbanowicz et al. 2012) and simulate varying degrees of detection difficulty (Urbanowicz et al. 2012a, b). Although effective, it is not always easy to tie probability-based simulation methods to biological concepts. To address this concern, methods such as Heuristic Identification of Biological Architectures for simulating Complex Hierarchical Interactions (HIBACHI) and associated software can be considered (Moore et al. 2015). The approach facilitates the development of mathematical models of epistasis based on biological mechanisms such as gene transcription and protein–protein interactions. Biology-based approaches may facilitate the development of epistasis modelling methods that are easier to interpret. Standard operating procedures are underway to replicate and validate GWAI results, along with appropriate definitions for these. We need better experimental methods for confirming statistical models of epistasis in animal models or in human cell culture.

Finally, moving from localization to function will be essential to explain molecular mechanisms playing a synergetic role in human complex diseases. Although there is still a long way to go before epistasis findings can be brought into clinical practice, our practical and theoretical experience has shown that, by taking advantage of various methodologies and by examining data from different angles, it is feasible to reveal strong evidence for biological gene interactions derived from genome-wide SNP panels.

## References

[CR1] Abegaz F, Van Lishout F, Mahachie John JM, Chaichoompu K, Bhardwaj A, Gusareva E, Wei Z, Hakonarsson H, Van Steen K (2018) Epistasis detection using model-based multifactor dimensionality reduction in structured populations. bioRxiv 541946

[CR2] Alanis-Lobato G, Cannistraci CV, Eriksson A, Manica A, Ravasi T (2015). Highlighting nonlinear patterns in population genetics datasets. Sci Rep.

[CR3] Bateson W (1907). Facts limiting the theory of heredity. Science.

[CR4] Belsley DA, Kuh E, Welsch RE (1980). Regression diagnostics: identifying influential data and sources of collinearity.

[CR5] Benjamini Y, Yekutieli D (2001). The control of the false discovery rate in multiple testing under dependency. Ann Stat.

[CR6] Bessonov K, Gusareva ES, Van Steen K (2015). A cautionary note on the impact of protocol changes for genome-wide association SNP x SNP interaction studies: an example on ankylosing spondylitis. Hum Genet.

[CR7] Box GEP, Cox DR (1964). An analysis of transformations. J R Stat Soc Ser B.

[CR8] Bradley JV (1978). Robustness?. Br J Math Stat Psychol.

[CR9] Cattaert T, Calle ML, Dudek SM, Mahachie John JM, Van Lishout F, Urrea V, Ritchie MD, Van Steen K (2011). A detailed view on model-based multifactor dimensionality reduction for detecting gene-gene interactions in case-control data in the presence of noise. Ann Hum Genet.

[CR10] Collins RL, Hu T, Wejse C, Sirugo G, Williams SM, Moore JH (2013). Multifactor dimensionality reduction reveals a three-locus epistatic interaction associated with susceptibility to pulmonary tuberculosis. BioData Min.

[CR11] Cordell HJ (2002). Epistasis: what it means, what it does not mean, and statistical methods to detect it in humans. Hum Mol Genet.

[CR12] Cordell HJ (2009). Detecting gene-gene interactions that underlie human diseases. Nat Rev Genet.

[CR13] Culverhouse R, Suarez BK, Lin J, Reich T (2002). A perspective on epistasis: limits of models displaying no main effect. Am J Hum Genet.

[CR14] de Leeuw CA, Neale BM, Heskes T, Posthuma D (2016). The statistical properties of gene-set analysis. Nat Rev Genet.

[CR15] Dering C, Konig IR, Ramsey LB, Relling MV, Yang W, Ziegler A (2014). A comprehensive evaluation of collapsing methods using simulated and real data: excellent annotation of functionality and large sample sizes required. Front Genet.

[CR16] Dohoo IR, Ducrot C, Fourichon C, Donald A, Hurnik D (1996). An overview of techniques for dealing with large numbers of independent variables in epidemiologic studies. Prev Vet Med.

[CR17] Duraisingh MT, Refour P (2005). Multiple drug resistance genes in malaria— from epistasis to epidemiology. Mol Microbiol.

[CR18] Ebbert MTW, Ridge PG, Kauwe JSK (2015). Bridging the gap between statistical and biological epistasis in Alzheimer’s disease. BioMed Res Int.

[CR19] Evans DM, Spencer CC, Pointon JJ, Su Z, Harvey D, Kochan G, Oppermann U, Dilthey A, Pirinen M, Stone MA (2011). Interaction between ERAP1 and HLA-B27 in ankylosing spondylitis implicates peptide handling in the mechanism for HLA-B27 in disease susceptibility. Nat Genet.

[CR20] Fisher RA (1918). The correlation between relatives on the supposition of Mendelian inheritance. Trans R Soc Edin.

[CR21] Fouladi R, Bessonov K, Van Lishout F, Van Steen K (2015). Model-based multifactor dimensionality reduction for rare variant association analysis. Hum Hered.

[CR22] Frånberg M, Gertow K, Hamsten A, Consortium PROCARDIS, Lagergren J, Sennblad B (2015). Discovering genetic interactions in large-scale association studies by stage-wise likelihood ratio tests. PLoS Genet.

[CR23] Friedman J (1991). Multivariate adaptive regression splines. Ann Stat.

[CR24] Glantz SA, Slinker BK (1990). Primer of applied regression and analysis of variance.

[CR25] Goeman JJ, Solari A (2014). Multiple hypothesis testing in genomics. Stat Med.

[CR26] Gola D, König IR (2016). Identification of interactions using model-based multifactor dimensionality reduction. BMC Proc.

[CR27] Gonzalez-Dominguez J, Wienbrandt L, Kassens JC, Ellinghaus D, Schimmler M, Schmidt B (2015). Parallelizing epistasis detection in GWAS on FPGA and GPU-accelerated computing systems. IEEE/ACM Trans Comput Biol Bioinform.

[CR28] Greene CS, Penrod NM, Kiralis J, Moore JH (2009). Spatially uniform relieff (SURF) for computationally-efficient filtering of gene-gene interactions. BioData Min.

[CR29] Greene CS, Penrod NM, Williams SM, Moore JH (2009). Failure to replicate a genetic association may provide important clues about genetic architecture. PLoS One.

[CR30] Greene CS, Sinnott-Armstrong NA, Himmelstein DS, Park PJ, Moore JH, Harris BT (2010). Multifactor dimensionality reduction for graphics processing units enables genome-wide testing of epistasis in sporadic ALS. Bioinformatics.

[CR31] Greene CS, Himmelstein DS, Nelson HH, Kelsey KT, Williams SM, Andrew AS, Karagas MR, Moore JH: Enabling personal genomics with an explicit test of epistasis. Pac Symp Biocomput 2010:327–336 10.1142/9789814295291_0035 PMC2916690 19908385

[CR32] Greenland S, Rothman KJ (1998). Concepts of interaction..

[CR33] Guerrero VM, Johnson RA (1982). Use of the Box-Cox transformation with binary response models. Biometrika.

[CR34] Gundlach S, Kässens JC, Wienbrandt L (2016). Genome-wide association interaction studies with MB-MDR and maxT multiple testing correction on FPGAs. Procedia Comput Sci.

[CR35] Gusareva ES, Van Steen K (2014). Practical aspects of genome-wide association interaction analysis. Hum Genet.

[CR36] Gusareva ES, Carrasquillo MM, Bellenguez C, Cuyvers E, Colon S, Graff-Radford NR, Petersen RC, Dickson DW, Mahachie John JM, Bessonov K (2014). Genome-wide association interaction analysis for Alzheimer’s disease. Neurobiol Aging.

[CR37] Gusareva E, Twizere JC, Sleegers K, Dourlen P, Abisambra JF, Meier S, Cloyd R, Weiss B, Dermaut B, Bessonov K (2018). Male-specific epistasis between WWC1 and TLN2 genes is associated with Alzheimer’s disease. Neurobiol Aging.

[CR38] Guyon I, Elisseeff A (2003). An introduction to variable selection and feature selection. J Mach Learn Res.

[CR39] Hu X, Liu Q, Zhang Z, Li Z, Wang S, He L, Shi Y (2010). SHEsisEpi, a GPU-enhanced genome-wide SNP-SNP interaction scanning algorithm, efficiently reveals the risk genetic epistasis in bipolar disorder. Cell Res.

[CR40] Hu T, Chen Y, Kiralis JW, Moore JH (2013). ViSEN: methodology and software for visualization of statistical epistasis networks. Genet Epidemiol.

[CR41] Hu T, Andrew AS, Karagas MR, Moore JH: Statistical epistasis networks reduce the computational complexity of searching three-locus genetic models. Pac Symp Biocomput 2013:397–408 PMC3587773 23424144

[CR42] Jorgenson E, Witte JS (2006). A gene-centric approach to genome-wide association studies. Nat Rev Genet.

[CR43] Kam-Thong T, Czamara D, Tsuda K, Borgwardt K, Lewis CM, Erhardt-Lehmann A, Hemmer B, Rieckmann P, Daake M, Weber F (2011). EPIBLASTER-fast exhaustive two-locus epistasis detection strategy using graphical processing units. Eur J Hum Genet.

[CR44] Kam-Thong T, Azencott CA, Cayton L, Putz B, Altmann A, Karbalai N, Samann PG, Scholkopf B, Muller-Myhsok B, Borgwardt KM (2012). GLIDE: GPU-based linear regression for detection of epistasis. Hum Hered.

[CR45] Kim MK, Moore JH, Kim JK, Cho KH, Cho YW, Kim YS, Lee MC, Kim YO, Shin MH (2011). Evidence for epistatic interactions in antiepileptic drug resistance. J Hum Genet.

[CR46] Knol MJ, VanderWeele TJ (2012). Recommendations for presenting analyses of effect modification and interaction. Int J Epidemiol.

[CR47] Lareau CA, McKinney BA (2015). Network theory for data-driven epistasis networks. Methods Mol Biol.

[CR48] Lehner B (2011). Molecular mechanisms of epistasis within and between genes. Trends Genet.

[CR49] Li W, Reich J (2000). A complete enumeration and classification of two-locus disease models. Hum Hered.

[CR50] Li J, Malley JD, Andrew AS, Karagas MR, Moore JH (2016). Detecting gene–gene interactions using a permutation-based random forest method. BioData Min.

[CR51] Liekens AM, De Knijf J, Daelemans W, Goethals B, De Rijk P, Del-Favero J (2011). BioGraph: unsupervised biomedical knowledge discovery via automated hypothesis generation. Genome Biol.

[CR52] Liu Y, Koyuturk M, Barnholtz-Sloan JS, Chance MR (2012). Gene interaction enrichment and network analysis to identify dysregulated pathways and their interactions in complex diseases. BMC Syst Biol.

[CR53] Mahachie John JM, Van Lishout F, Van Steen K (2011). Model-based multifactor dimensionality reduction to detect epistasis for quantitative traits in the presence of error-free and noisy data. Eur J Hum Genet.

[CR54] Mahachie John JM, Cattaert T, De Lobel L, Van Lishout F, Empain A, Van Steen K (2011). Comparison of genetic association strategies in the presence of rare alleles. BMC Proc.

[CR55] Martin ER, Tunc I, Liu Z, Slifer SH, Beecham AH, Beecham GW (2018). Properties of global- and local-ancestry adjustments in genetic association tests in admixed populations. Genet Epidemiol.

[CR56] Montojo J, Zuberi K, Rodriguez H, Bader GD, Morris Q (2014). GeneMANIA: fast gene network construction and function prediction for cytoscape. F1000Res.

[CR57] Moore JH (2015). Epistasis analysis using ReliefF. Methods Mol Biol.

[CR58] Moore JH, Hu T (2015). Epistasis analysis using information theory. Methods Mol Biol.

[CR59] Moore JH, Williams SM (2005). Traversing the conceptual divide between biological and statistical epistasis: systems biology and a more modern synthesis. Bioessays.

[CR60] Moore JH, Williams SM (2009). Epistasis and its implications for personal genetics. Am J Hum Genet.

[CR61] Moore JH, Gilbert JC, Tsai CT, Chiang FT, Holden T, Barney N, White BC (2006). A flexible computational framework for detecting, characterizing, and interpreting statistical patterns of epistasis in genetic studies of human disease susceptibility. J Theor Biol.

[CR62] Moore JH, Amos R, Kiralis J, Andrews PC (2015). Heuristic identification of biological architectures for simulating complex hierarchical genetic interactions. Genet Epidemiol.

[CR63] Moskvina V, Schmidt KM (2008). On multiple-testing correction in genome-wide association studies. Genet Epidemiol.

[CR64] Nicodemus KK, Liu W, Chase GA, Tsai YY, Fallin MD (2005). Comparison of type I error for multiple test corrections in large single-nucleotide polymorphism studies using principal components versus haplotype blocking algorithms. BMC Genet.

[CR65] North BV, Curtis D, Sham PC (2005). Application of logistic regression to case-control association studies involving two causative loci. Hum Hered.

[CR66] Norton B, Pearson ES (1976). A note on the background to and refereeing of R.A. Fisher’s 1918 paper ‘The correlation between relatives on the supposition of Mendelian inheritance’. Notes Rec R Soc Lond.

[CR67] Nyholt DR (2004). A simple correction for multiple testing for single-nucleotide polymorphisms in linkage disequilibrium with each other. Am J Hum Genet.

[CR68] Park MY, Hastie T (2008). Penalized logistic regression for detecting gene interactions. Biostatistics.

[CR69] Phillips PC (1998). The language of gene interaction. Genetics.

[CR70] Phillips PC (2008). Epistasis—the essential role of gene interactions in the structure and evolution of genetic systems. Nat Rev Genet.

[CR71] Piette ER, Moore JH (2017). Improving the reproducibility of genetic assoication results using genotype resampling methods. Lect Notes Comput Sci.

[CR72] Piriyapongsa J, Ngamphiw C, Intarapanich A, Kulawonganunchai S, Assawamakin A, Bootchai C, Shaw PJ, Tongsima S (2012). iLOCi: a SNP interaction prioritization technique for detecting epistasis in genome-wide association studies. BMC Genom.

[CR73] Purcell S, Neale B, Todd-Brown K, Thomas L, Ferreira MA, Bender D, Maller J, Sklar P, de Bakker PI, Daly MJ, Sham PC (2007). PLINK: a tool set for whole-genome association and population-based linkage analyses. Am J Hum Genet.

[CR74] Putz B, Kam-Thong T, Karbalai N, Altmann A, Muller-Myhsok B (2013). Cost-effective GPU-grid for genome-wide epistasis calculations. Methods Inf Med.

[CR75] Ritchie MD (2011). Using biological knowledge to uncover the mystery in the search for epistasis in genome-wide association studies. Ann Hum Genet.

[CR76] Ritchie MD, Van Steen K (2018). The search for gene-gene interactions in genome-wide association studies: challenges in abundance of methods, practical considerations, and biological interpretation. Ann Transl Med.

[CR77] Ritchie MD, Hahn LW, Roodi N, Bailey LR, Dupont WD, Parl FF, Moore JH (2001). Multifactor-dimensionality reduction reveals high-order interactions among estrogen-metabolism genes in sporadic breast cancer. Am J Hum Genet.

[CR78] Sackton TB, Hartl DL (2016). Genotypic context and epistasis in individuals and populations. Cell.

[CR79] Satagopan JM, Elston RC (2013). Evaluation of removable statistical interaction for binary traits. Stat Med.

[CR80] Shannon P, Markiel A, Ozier O, Baliga NS, Wang JT, Ramage D, Amin N, Schwikowski B, Ideker T (2003). Cytoscape: a software environment for integrated models of biomolecular interaction networks. Genome Res.

[CR81] Slim L, Chatelain C, Azencott CA, Vert J-P (2018) Novel methods for epistasis detection in genome-wide association studies. bioRxiv 442749 10.1371/journal.pone.0242927 PMC7703915 33253293

[CR82] Sun W, Cai TT (2009). Large-scale multiple testing under dependence. J R Statist Soc B (2009).

[CR83] Sun X, Lu Q, Mukherjee S, Crane PK, Elston R, Ritchie MD (2014). Analysis pipeline for the epistasis search-statistical versus biological filtering. Front Genet.

[CR84] Ueki M, Cordell HJ (2012). Improved statistics for genome-wide interaction analysis. PLoS Genet.

[CR85] Urbanowicz RJ, Kiralis J, Fisher JM, Moore JH (2012). Predicting the difficulty of pure, strict, epistatic models: metrics for simulated model selection. BioData Min.

[CR86] Urbanowicz RJ, Kiralis J, Sinnott-Armstrong NA, Heberling T, Fisher JM, Moore JH (2012). GAMETES: a fast, direct algorithm for generating pure, strict, epistatic models with random architectures. BioData Min.

[CR87] Van Steen K (2012). Travelling the world of gene-gene interactions. Brief Bioinform.

[CR88] Van Steen K, Malats N (2015) Perspectives on data integration in human complex disease analysis. In: Big data analytics in bioinformatics and healthcare. IGI GLobal, pp 284–322. https://www.igi-global.com/chapter/perspectives-on-data-integration-in-human-complex-disease-analysis/121463

[CR89] Van Steen K, Molenberghs G (2012) Multicollinearity. In: Chow S-C (ed) Encyclopedia of biopharmaceutical statistics, 3rd edn

[CR90] Van Steen K, McQueen MB, Herbert A, Raby B, Lyon H, Demeo DL, Murphy A, Su J, Datta S, Rosenow C (2005). Genomic screening and replication using the same data set in family-based association testing. Nat Genet.

[CR91] Van Lishout F, Mahachie John JM, Gusareva ES, Urrea V, Cleynen I, Theatre E, Charloteaux B, Calle ML, Wehenkel L, Van Steen K (2013). An efficient algorithm to perform multiple testing in epistasis screening. BMC Bioinformatics.

[CR92] Van Lishout F, Gadaleta F, Moore JH, Wehenkel L, Van Steen K (2015). gammaMAXT: a fast multiple-testing correction algorithm. BioData Min.

[CR93] VanderWeele TJ (2009). On the distinction between interaction and effect modification. Epidemiology.

[CR94] VanderWeele TJ, Laird NM (2011). Tests for compositional epistasis under single interaction-parameter models. Ann Hum Genet.

[CR95] Vansteelandt S, Vanderweele TJ, Robins JM (2008). Multiply robust inference for statistical interactions. J Am Stat Assoc.

[CR96] Vansteelandt S, Bekaert M, Claeskens G (2012). On model selection and model misspecification in causal inference. Stat Methods Med Res.

[CR97] Vermeulen SH, Den Heijer M, Sham P, Knight J (2007). Application of multi-locus analytical methods to identify interacting loci in case-control studies. Ann Hum Genet.

[CR98] Wan X, Yang C, Yang Q, Xue H, Fan X, Tang NL, Yu W (2010). BOOST: A fast approach to detecting gene-gene interactions in genome-wide case-control studies. Am J Hum Genet.

[CR99] Wang X, Elston RC, Zhu X (2010). The meaning of interaction. Hum Hered.

[CR100] Wang Z, Wang Y, Tan KL, Wong L, Agrawal D (2011). eCEO: an efficient cloud epistasis computing model in genome-wide association study. Bioinformatics.

[CR101] Wang MH, Sun R, Guo J, Weng H, Lee J, Hu I, Sham PC, Zee BC (2016). A fast and powerful W-test for pairwise epistasis testing. Nucleic Acids Res.

[CR102] Wei WH, Hemani G, Haley CS (2014). Detecting epistasis in human complex traits. Nat Rev Genet.

[CR103] Westfall PH, Young SS (1993). Resampling-base multiple testing.

[CR104] Wilson BA, Garud NR, Feder AF, Assaf ZJ, Pennings PS (2016). The population genetics of drug resistance evolution in natural populations of viral, bacterial and eukaryotic pathogens. Mol Ecol.

[CR105] Winham SJ, Colby CL, Freimuth RR, Wang X, de Andrade M, Huebner M, Biernacka JM (2012). SNP interaction detection with random forests in high-dimensional genetic data. BMC Bioinform.

[CR106] Wong AK, Krishnan A, Yao V, Tadych A, Troyanskaya OG (2015). IMP 2.0: a multi-species functional genomics portal for integration, visualization and prediction of protein functions and networks. Nucleic Acids Res.

[CR107] Zhang Q (2015). Associating rare genetic variants with human diseases. Front Genet.

[CR108] Zhang F, Boerwinkle E, Xiong M (2014). Epistasis analysis for quantitative traits by functional regression model. Genome Res.

